# Syncope in the Emergency Department: A Case Report of a Rare Presentation of Favism

**DOI:** 10.7759/cureus.64592

**Published:** 2024-07-15

**Authors:** Céline Couvreur, John Mitchell, Patrice Forget, Henri Thonon

**Affiliations:** 1 Emergency Medicine, CHU (Centre Hospitalier Universitaire) UCLouvain (Université Catholique de Louvain) Namur - Godinne, Godinne, BEL; 2 Anesthesiology, CHU (Centre Hospitalier Universitaire) UCLouvain (Université Catholique de Louvain) Namur - Godinne, Godinne, BEL; 3 Anesthesiology, University of Aberdeen, Aberdeen, GBR

**Keywords:** haemoglobinemia, hemighosts, syncope, favism, g6pd deficiency

## Abstract

Consumption of fava beans in a patient with glucose-6-phosphate dehydrogenase (G6PD) deficiency, also called favism, can lead to a haemolytic crisis. We report the case of a 69-year-old patient of Iranian origin admitted to the emergency department following syncope. The patient's comprehensive interview and blood analysis revealed that the patient presented a haemolytic crisis triggered by fava beans consumption, due to previously undiagnosed G6PD deficiency. The pathophysiology of favism is complex and clinical presentations of G6PD deficiency are numerous due to multiple genetic variants. Indirect signs, such as the presence of methemoglobinaemia and hemighosts on the blood smear, can aid in the diagnosis. This case highlights the importance of considering G6PD deficiency as a potential diagnosis in case of haemolytic crisis, even in elderly patients.

## Introduction

Glucose-6-phosphate dehydrogenase (G6PD) is an enzyme involved in the protection of red blood cells against oxidative stresses, the most common being infection [1]. The prevalence of G6PD deficiency is estimated at more than 350 million individuals worldwide [2], with a predominance of males [3]. It is most commonly found in Africa, Asia, the Mediterranean basin, and the Middle East.

Most patients with G6PD deficiency are asymptomatic but may present with haemolytic crises, particularly when eating fava beans. This is why G6PD deficiency is better known as favism. The manifestations of a haemolytic crisis in patients with favism are diverse, ranging from fatigue to syncope [4].

We report the case of a patient admitted to the emergency department following syncope due to a haemolytic crisis in a context of favism, unaware of having G6PD deficiency. The aim of this article is to review the different clinical presentations of a haemolytic crisis, as well as diagnostic and therapeutic approaches.

## Case presentation

A 69-year-old male of Iranian origin was admitted to the emergency department for syncope. At home, the patient suffered from loss of consciousness preceded by dizziness, resulting in mild head trauma. On admission, the patient reported generalized fatigue and mild abdominal pain for the past 24 hours. The patient's history included Brugada syndrome, for which he had an internal defibrillator, hypercholesterolemia and hypertension. His chronic treatment included perindopril and rosuvastatin-ezetimibe. His clinical examination showed blood pressure measurements of 100/60 mmHg, tachycardia at 110 beats per minute, temperature at 37.9°C, and pulsed oxygen saturation at 90% on room air. His Glasgow Coma Scale (GCS) on admission was 15/15.

**Figure 1:**
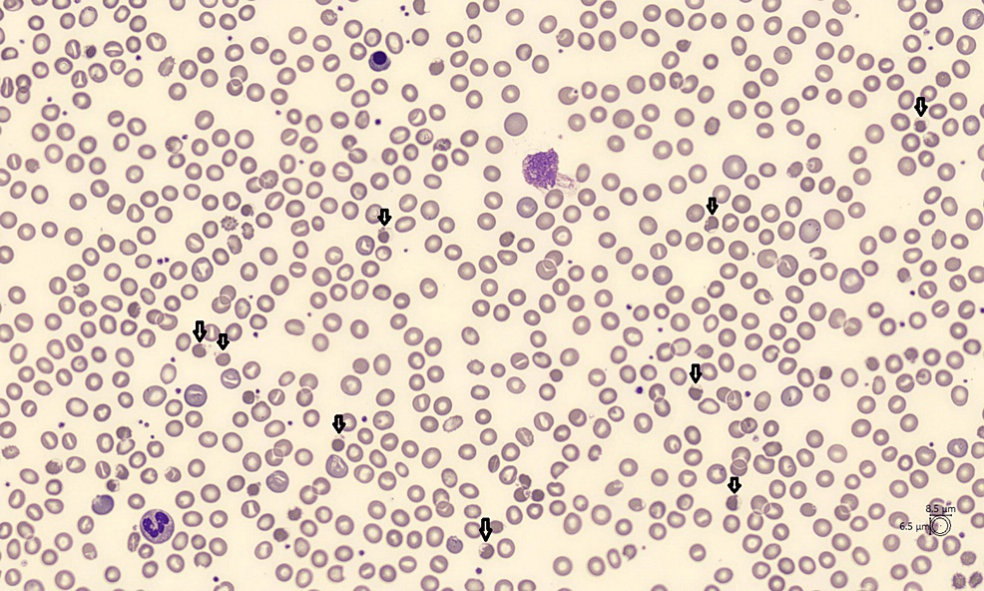
Patient's blood smear with multiple red blood cells partially emptied of their contents, called hemighosts

The patient presented with mucocutaneous jaundice. An analysis of potential arrhythmia recorded in his defibrillator was requested and showed no arrhythmia concomitant with the syncope. Arterial blood gases were collected on admission and showed arterial oxygen pressure of 73 mmHg and arterial oxygen saturation of 95%. There was therefore a slight discrepancy between the oxygen saturation measured by oximeter and by gasometry. This blood gas analysis also showed a methaemoglobin level of 3% (for a norm below 2%). Blood biology results are presented in Table 1. Blood smear revealed phantom red blood cells called hemighosts (Figure 1).

**Table 1:** Patient’s blood biology results

Dosage	Result	Reference Range
C-reactive protein (CRP)	70 mg/L	< 5mg/L
Haemoglobin	11.1 g/dL	13.3-16.7 g/dL
Reticulocytes	4.9%	0.5-2%
Creatinine	0.7 mg/dL	< 1.2 mg/dL
Total bilirubin	6.6 mg/dL	< 1.3 mg/dL
Unconjugated bilirubin	6.0 mg/dL	<0.8 mg/dL
Lactate dehydrogenase (LDH)	707 U/L	< 250 U/L
Haptoglobin	0.1 g/L	0.3-2.0 g/L

The Coombs test for haemolytic antibodies was negative. Basic bacteriological examination, including chest X-ray, urine sediment and molecular biology test for respiratory viruses, was negative. The patient described no recent medication changes but did report consumption of fava beans 48 hours prior to admission. The patient reported having consumed fava beans several times in the past without developing symptoms and that he was not known to have G6PD deficiency, in contrast with the current syncope in the context of a haemolytic crisis due to favism.

He was admitted to a general internal medicine unit for monitoring. In the emergency department, given the presence of haemolytic anaemia with hemighosts, the patient's ethnic origin, and the recent consumption of fava beans, a genetic analysis for G6PD deficiency was requested. The results, which came a few days later, showed a partial deficiency. His haemoglobin level dropped to 7.9 g/dL but did not require any red blood cell transfusion; the patient only received an oral folate supplement. After one week, the haemoglobin level was 8.5 g/dL and the patient was discharged with recommendations to avoid beans and certain medications.

## Discussion

G6PD is an enzyme present in every animal cell. It catalyzes the first step of the pentose pathway, one of the four main pathways of energy metabolism. In a red blood cell, the pentose pathway allows the production of nicotinamide adenine dinucleotide phosphate (NADPH). NADPH is essential for maintaining glutathione in its reduced form, the only one allowing the red blood cell to defend itself against oxidative stress linked to the production of free radicals [1].

Significant oxidative stress can be induced by certain medications, infectious diseases or the consumption of certain foods, particularly fava beans. Medications to avoid in patients with favism include primaquine, sulfamethoxazole, nitrofurantoin, and methylene blue. The infectious agents most frequently involved in a haemolytic crisis are hepatitis A and B viruses, cytomegalovirus, any bacteria responsible for pneumonia, and *Salmonella typhi.* In addition to fava beans, quinine-based drinks and dietary supplements containing vitamin C are also prohibited [5]. Fava beans contain two beta-glucosides that trigger haemolysis: vicine and convicine. Once ingested, these two molecules are converted in the intestine, leading to increased production of free radicals. These free radicals are responsible for oxidative stress, increasing glutathione oxidation and making red blood cells more vulnerable [4].

The severity of symptoms is related to the amount of beta-glucosides ingested and depends on the quantity of beans, the type of preparation, the degree of maturity of the beans and patient-specific factors such as age [6,7]. Fresh beans contain more beta-glucosides than dried beans due to their green shell [8]. Soaking before cooking reduces the vicine and convicine contents by 56% and 34%, respectively. Cooking has little impact on the level of beta-glucosides [9]. These factors explain why the diagnosis of mild to moderate G6PD deficiency is sometimes made at an advanced age, following a first attack; severe forms being diagnosed in paediatrics.

Genetically, G6PD is an X-linked enzyme, resulting in male predominance. Females can be heterozygous since they have two copies of the G6PD gene; homozygous forms in populations with a high prevalence are possible. Due to the genetic mosaicism resulting from X chromosome inactivation, heterozygous females can be as deficient in G6PD as males [5]. To date, 217 G6PD genetic variants have been identified and classified by the World Health Organisation from class I to IV, ranging from the most severe to the mildest forms. Old age is not an exclusion criterion for the diagnosis of favism; mild forms can remain minimally symptomatic, unlike severe forms discovered in childhood [10]. Patient's ethnic origin is essential information, as these mutations are mainly found in Africa, Asia, the Mediterranean basin, and the Middle East [11]. Screening campaigns are organised in high-prevalence regions. Screening is carried out mainly in patients who have suffered from a first episode of haemolysis and in icteric newborns. The aim of such screening is to prevent a major crisis by avoiding high-risk foods or drugs. Rapid testing has become possible, particularly in developing countries, thanks to the advent of simpler molecular methods such as polymerase chain reaction (PCR) [6]. In 2009, Peters et al. also described a correlation between the prevalence of G6PD deficiency and the distribution of malaria, suggesting that G6PD deficiency provides protection against infection [12].

In a patient suffering from favism, symptoms begin between 24 hours and five days after eating fava beans. The main symptoms of a haemolytic crisis or acute haemolytic anaemia are fatigue and abdominal or back pain. A sub-febrile state is sometimes present. To our knowledge, this is the second described case of syncope as the inaugural symptom of a haemolytic crisis on favism; the first case having been described by Soyuncu et al. in 2010 [13]. Syncope is defined as a short-duration loss of consciousness, caused by a transient decrease in cerebral blood flow [14]. The hypothesis is that syncope is secondary to a drop in cerebral oxygenation caused by a rapid drop in haemoglobin level associated with a state of fever-induced dehydration.

Biologically, classic markers of haemolysis are always present: regenerative anaemia, unconjugated hyperbilirubinemia, low haptoglobin, and increased lactate dehydrogenase (LDH). This hyperbilirubinemia induces dark urine [6]. Low to moderate methaemoglobinaemia is frequently found chronically in patients with favism [4]. Methaemoglobin is a form of haemoglobin in which the iron cation is in an oxidized state (ferric rather than ferrous), and therefore unable to transport oxygen. One of the physiological mechanisms for reducing methaemoglobin to haemoglobin is that of NADPH-dependent methaemoglobin reductase [15]. As its name suggests, this enzyme depends on the production of NADPH, which is lacking in G6PD-deficient patients. In methaemoglobinaemia, as in other haemopathies, a dissociation between pulsed oxygen saturation and arterial oxygen saturation can be observed [16]. In our patient, a slight dissociation was observed. During a crisis, microscopic observation of blood cells reveals phantom red blood cells, also called (hemi)ghosts. These are red blood cells partially emptied of their haemoglobin content [17].

From a therapeutic point of view, there is little literature on the subject. In most cases of favism, symptoms are moderate and transient, and treatment is symptomatic. Avoidance of the triggering factor and supporting red blood cell regeneration through oral administration of vitamins B9-12 and iron would be the only treatment in most cases. In more severe forms, red blood cell transfusions may be necessary. The most severe and common complication is acute renal failure. This is the result of acute tubular necrosis due to ischemia and tubular obstruction by haemoglobin casts [5] and sometimes requiring dialysis [4]. Fluid repletion and alkalinisation can prevent haemolysis-induced renal failure [18]. Paracetamol in case of fever is authorized but should not exceed the recommended doses. Cases of severe haemolysis due to paracetamol overdose have been described [5].

## Conclusions

Haemolytic crises due to favism may be responsible for atypical presentations such as syncope. The diagnosis is based on patient’s history regarding their ethnic origin and diet, as well as paraclinical examinations looking for methaemoglobin and hemighosts. This diagnosis must not be forgotten, even in elderly patients.
